# Improving precision for detecting change in the shape of the cornea in patients with keratoconus

**DOI:** 10.1038/s41598-018-30173-7

**Published:** 2018-08-17

**Authors:** Matthias Brunner, Gabriela Czanner, Riccardo Vinciguerra, Vito Romano, Sajjad Ahmad, Mark Batterbury, Claire Britten, Colin E. Willoughby, Stephen B. Kaye

**Affiliations:** 1 Department of Corneal and External Eye Diseases, St. Paul’s Eye Unit, Royal Liverpool University Hospital, Liverpool, United Kingdom; 2 Department of Eye and Vision Science, University of Liverpool, Liverpool, United Kingdom; 3 Department of Surgical Sciences, Division of Ophthalmology, University of Insubria, Varese, Italy; 4 Department of Biostatistics, University of Liverpool, Liverpool, United Kingdom

## Abstract

To investigate a method for precision analysis to discriminate true corneal change from measurement imprecision in keratoconus (KC). Thirty patients with KC and 30 healthy controls were included. Coefficients of repeatability and limits of agreement (LOA) were compared using multiple measurements for inter-observer and inter-device agreement with the Pentacam HR, Orbscan IIz, and Tomey Casia SS-1000. Correlation of repeated measurements was evaluated using a linear mixed effect model (also called random effect model). A formula was derived for the theoretical expected change in precision and compared with measured change. Correlation between measurements from the same eye was small (R = 0.13). The 99.73% LOA (3 SD) of the mean of three measurements, provided better precision than 95% LOA (2 SD) of single cut-off values as expected from statistical theory for uncorrelated measurements for evidence of a significant change in corneal shape in patients with keratoconus. This enabled the determination of cut-off values for the detection of true change in corneal shape. The mean of three repeated measurements will provide better precision when there is minimal correlation. Three (rather than two) standard deviations provides a precise estimate of the LOA within or between observers and can be used as a reliable measure for identifying stage-independent corneal shape changes (progression) in keratoconus.

## Introduction

Keratoconus (KC) is a sight-threatening corneal condition, which is characterized by a usually bilateral progressive ectasia and thinning of the cornea. KC affects 265 to 2,300 out of 100,000 people^1,2^, and accounts for more than 25% of corneal transplants undertaken in Europe and the United States^3,4^.

Emerging new treatment strategies and imaging technology have improved the management of KC^5^. Corneal collagen cross-linking (CXL), which was clinically introduced in 2003^6^, is perhaps the most recent promising innovation in the treatment of KC^7^. Recent FDA approval in the United States in 2016 will likely have a further impact on treatment standards for KC worldwide. CXL can delay or halt further progression of KC and reduce the need for corneal transplantation^8^. Early detection of progressive disease is a crucial requisite when considering CXL.

The introduction of corneal topography and tomography has improved the ability to diagnose KC by increasing the ability to identify corneal ectatic change at an earlier stage than has been previously possible^9^. Despite advances in the field of imaging technology, measurement imprecision remains an important issue in identifying and discriminating change as a marker of disease progression.

Calibration and validation of clinical instrument precision is usually established in normal healthy controls. For pathologic corneal conditions, these normative limits may not be valid.

Several studies have demonstrated better measurement precision in healthy eyes^10,11^ than keratoconic eyes^12–14^, for various clinical parameters and different imaging devices. Previously reported repeatability and reproducibilty data of keratoconic eyes have been inconsistent and vary considerably among different studies and devices. Furthermore, measurement precision has been reported to be dependent on the stage of KC with a decrease of precision in more advanced disease^13,15^, which renders early detection of corneal shape change in progressive disease difficult.

Most studies that have investigated the precision of corneal topography and tomography devices, have derived their precision analysis from two single measurements with little consideration to multiple measurements. Although it is well understood that precision increases when a mean of several measurements is used, what is less known, is that the increase in precison is largest for uncorrelated measurements (by a factor of √p, where p is the number of measurements), while in contrast, there is no increase in precision for perfectly correlated measurements. To date there has been little research on measurement correlation and therefore the evidence for the improvement of precision is unclear. Although precision is often expressed as 95% limits of agreement (LOA) using 95% confidence intervals, using three standard deviations (99.7%) provides an opportunity to improve specificity. The aim of this study, therefore, was to propose and investigate a method for precision analysis to better discriminate change from measurement imprecision in patients with KC, and to propose more precise cut-off values for progression of disease.

## Methods

Patients with an established diagnosis of KC and healthy volunteers attending The Royal Liverpool University Hospital over a 6-month period between September 2015 and February 2016 were invited to participate. The study received Institutional Review Board approval of The Royal Liverpool University Hosptial and was conducted accordingly to the ethical standards set in the 1964 Declaration of Helsinki, as revised in 2000. All patients provided informed consent.

All participants underwent serial imaging on three devices (Pentacam HR, Orbscan IIz and Casia SS-1000) during the same visit. One eye of each subject had two sets of three repeated scans taken by two different observers on each device (total of 18 consecutive scans per eye). The order of devices and observers performing the scans was random and patients had a short break between each set of measurements. The manufacturer’s instructions were followed by all operators and all measurements were made in scotopic luminance.

Investigated parameters included keratometric (K) readings in the flat (K1) and steep meridians (K2), corneal thickness at the thinnest location (TCT), and the maximum K reading (Kmax, Pentacam HR only). For the KC group, the worse eye of patients with untreated bilateral KC stage 1 to 3 using the Topographic Keratoconus Classification (TKC)^16^ was included. For the control group, one random eye of healthy volunteers was included if a Belin/Ambrósio Enhanced Ectasia total deviation index (BAD-D) of less than 1.6 SD from normative values indicated absence of ectasia in both eyes^17,18^. Exclusion criteria included the following: TKC stage 4 (massive scarring or thinning) in eyes with keratoconus, any additional ocular disease or drop use, systemic disease with potential corneal involvement, any previous ocular or refractive surgery, high myopia (>10 D) in the control group, contact lens wear, inability to fixate on a target or any type of continuous eye movements and nystagmus, and age below 18 years.

### Devices

The Pentacam HR (Oculus GmbH, Wetzlar, Germany) uses a Scheimpflug camera that performs a corneal scan by rotating around the optical axis from zero to 180° along with a monochromatic 475 nm UV-free slit-light source. The camera captures from 25 to 100 slit images depending on user settings^19^. All scans were performed in the 25-picture and automatic release mode.

The Orbscan IIz (Bausch & Lomb Surgical Inc, Rochester, New York) combines slit-scanning technology with a Placido disc system. The slit-scanning system directly measures anterior and posterior corneal surface elevations by performing two vertical scans through the projection of 40 optical slits (20 from the right and 20 from the left) onto the cornea at a fixed angle of 45 degrees to the instrument axis^19^. A 0.92 acoustic correction factor was applied as recommended by the manufacturer.

The Casia SS-1000 (Tomey Corp., Nagoya, Japan) uses 1310 nm high-speed swept-source OCT technology. Images are generated using a rate of 30 000 A-scans per second with a 10.0 mm diameter scanning range. For assessment of the corneal topography, 16 radial cross-sectional images are obtained in 0.34 seconds during 1 measurement^20^.

### Statistical Analysis

Summary statistics were used to describe the participant’s demographics and measurement readings as mean ± SD. The person who operated the imaging device was also responsible for storing the measurements and we refer to such person as an ‘observer’”. Bland–Altman analysis^21^ was performed to assess repeatability (intra-observer agreement) and reproducibility (inter-observer and inter-device agreement). Coefficients of repeatability were calculated for all means and expressed as ± 1.96 SD (95%) and ±3 SD (99.73%) limits of agreement (LOA) with 95% confidence intervals^21^. The level of agreement between two measurements was determined by the magnitude of its limits with lower values indicating better precision and *vice versa*. Intra-observer agreement (measurement variability in the same observer for the same device, *repeatability*) was calculated using the mean difference of two repeat measurements by the same observer. Inter-observer agreement (measurement precision between observers for the same device, *reproducibility*) was analysed using the first measurement and the mean of three repeat measurements from each of the two observers. Inter-device agreement (measurement precision between observers for different devices, *reproducibility*) was assessed using the first measurement and the mean of three repeat measurements from observer one. For both inter-observer and inter-device agreement, the coefficients of repeatability and LOAs were compared using the first measurement and the mean of three repeat measurements and were reported with the respective 95% confidence interval. Such calculation is equivalent to a linear mixed model with a random factor for the eye and a fixed factor for the observer for reproducibility between observers, and the device for the reproducibility between devices (see also *Supplementary Material, Reproducibility analysis*) for a general formula for multiple repeated measurements and details on the theoretical expected change in agreement). A correlation of the measurements from the same cornea was reported from a linear mixed model (also called random effect model). A p-value of less than 0.05 was considered to be statistically significant. The upper limit of the 95% confidence interval of the 99.73% (i.e. 3 SD) LOA was considered as the cut-off value for the progression in keratoconic eyes. The required sample size was estimated based on a reproducibility within-subject SD of 0.83 for Kmax in patients with mild to moderate KC, which corresponds to a SD of 1.17 D (0.83/$\surd 2$) for the difference of two measurements^22^. For the mean of 3 measurements, the SD of the difference of two means is expected to reduce to 0.68 D (1.17/$\surd 3$). If the maximum allowed difference between two means is 2.31 D, this can be confirmed with a sample size of 29 eyes^21^.

## Results

### Patient demographics

A total of sixty eyes of sixty patients were included: 30 healthy subjects and 30 patients with KC. The control group included 15 right eyes, 16 females and a mean age of 31.6 ± 10 years (median, 30; range, 18–58). The KC group comprised 17 right eyes, 12 females with a younger mean age of 26.7 ± 8 years (median, 26; range, 18–47) than the control gorup (p = 0.03). Sixteen eyes in the KC group were TKC stage 3 (53.4%), 10 eyes stage 2 (33.3%), and 4 eyes stage 1 (13.3%). The mean readings in healthy control and keratoconic eyes obtained with the Pentacam HR were 42.66 D and 45.70 D for K1, 43.50 D and 49.42 D for K2, 43.99 D and 56.53 D for Kmax, and 538.33 μm and 450.67 μm for TCT, respectively (p < 0.01 for all parameters). High quality scans (‘quality specification’ (QS) function determined as ‘OK’) were achieved in all healthy eyes (180 scans) and in 91.7% of the keratoconic eyes (165 scans). Corneal curvature values were significantly higher and thickness values significantly lower in the KC group (p < 0.01).

### Repeatability (intra-observer agreement)

**Figure 1:**
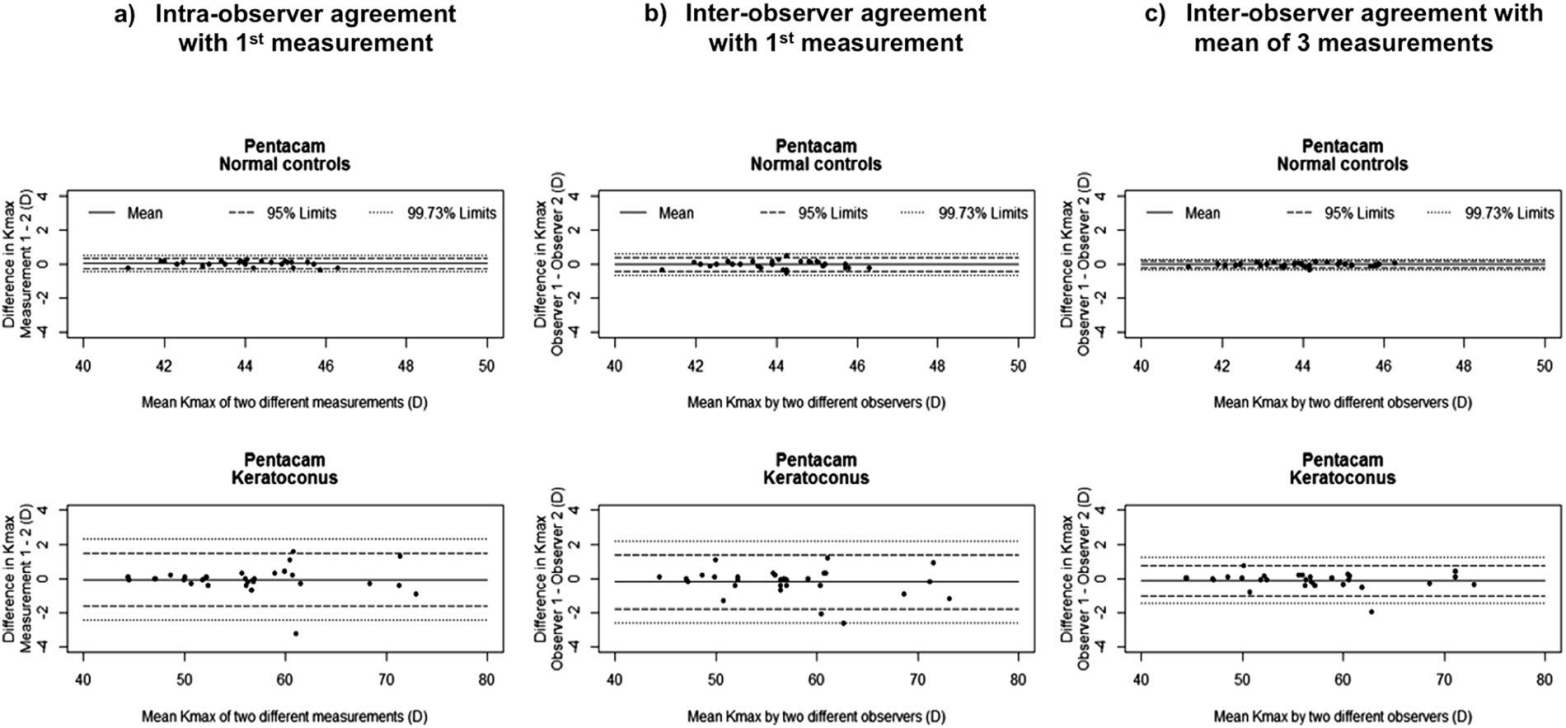
Bland-Altman plots for intra- and inter-observer 95% and 99.73% limits of agreement (LOA) for Kmax (maximal keratometry) in healthy and keratoconic eyes (n = 30).

Intra-observer agreements for K1, K2, TCT and Kmax (Pentacam) in healthy and keratoconic eyes, expressed as 95% and 97.7% LOA, are summarized in Table 1. Precision for all investigated parameters was better in healthy than keratoconic eyes, except for K1 readings with the Orbscan, which had similar upper LOA for both groups (0.99 D for healthy and 1.23 D for keratoconic eyes), and TCT readings with the Pentacam (11.37 D and 12.19 D, respectively). When comparing devices for keratoconic eyes, best intra-observer precision was seen with the Pentacam for keratometric readings (upper 99.73% LOA 0.74 D for K1 and 1.21 D for K2), and best precision for TCT was found with the Casia-SS1000 (upper 99.73% LOA in keratoconic eyes 15.30 μm). The upper 95% and 99.73% LOA for Kmax based on one measurement were 1.49 D and 2.32 D within observers (Fig. 1a). No significant measurement biases were found for any of the investigated parameters or devices (Supplementary Table S1 and Supplementary Figures S1–3).

**Table 1:** Intra- and inter-observer 95% and 99.73% limits of agreement (LOA) for K1, K2, and TCT with the Pentacam HR, Orbscan IIz, and Casia SS-1000 in healthy (n = 30) and keratoconic eyes (n = 30), using one or the mean of three repeat measurements.

	Device	Parameter	Intra-observer agreement			Inter-observer agreement					
			*1 Measurement*			*1 Measurement*			*Mean of 3 measurements*		
			SD	95% LOA	99.73% LOA	SD	95% LOA	99.73% LOA	SD	95% LOA	99.73% LOA
Healthy eyes	Pentacam HR	Kmax	0.15	0.35	0.51	0.21	0.41	0.63	0.11	0.20	0.31
		K1 (D)	0.08	0.15	0.24	0.09	0.19	0.29	0.04	0.08	0.12
		K2 (D)	0.09	0.17	0.27	0.13	0.23	0.37	0.07	0.11	0.18
		TCT (μm)	5.07	11.37	16.64	5.24	10.96	16.61	2.70	5.18	7.99
	Orbscan IIz	K1 (D)	0.21	0.99	1.58	0.59	0.99	1.61	0.30	0.61	0.82
		K2 (D)	0.5	0.9	1.41	0.40	0.7	1.11	0.23	0.36	0.59
		TCT (μm)	9.48	17.95	27.81	9.37	18.1	27.85	4.91	10.21	15.32
	Casia SS-1000	K1 (D)	0.17	0.34	0.52	0.22	0.43	0.66	0.09	0.19	0.28
		K2 (D)	0.19	0.37	0.57	0.22	0.44	0.67	0.10	0.29	0.35
		TCT (μm)	1.33	2.14	3.53	1.78	2.8	4.65	1.18	1.85	3.07
Keratoconus	Pentacam HR	Kmax	0.79	1.49	2.32	0.81	1.37	2.21	0.46	0.78	1.25
		K1 (D)	0.24	0.49	0.74	0.39	0.72	1.13	0.21	0.35	0.57
		K2 (D)	0.40	0.8	1.21	0.22	1.27	1.93	0.22	0.38	0.61
		TCT (μm)	5.9	12.19	18.33	6.98	13.71	20.97	3.39	6.4	9.93
	Orbscan IIz	K1 (D)	0.57	1.23	1.82	1.76	3.63	5.46	1.22	2.67	3.93
		K2 (D)	0.65	1.18	1.86	2.28	4.85	7.22	1.54	3.20	4.80
		TCT (μm)	26.63	46.03	73.72	27.40	53.08	81.58	17.24	33.73	51.65
	Casia SS-1000	K1 (D)	0.88	1.72	2.63	1.01	1.81	2.86	0.58	2.46	3.52
		K2 (D)	1.55	2.89	4.50	1.69	3.48	5.23	0.76	1.39	2.19
		TCT (μm)	4.98	10.13	15.3	9.9	21.70	31.99	7.08	14.69	22.06

LOA = Limits of agreement, SD = Standard deviation of difference of 2 measurements.

### Reproducibility

#### Inter-observer agreement

Precision between observers was better for healthy than keratoconic eyes with all devices. In keratoconic eyes, best inter-observer agreement for all investigated parameters was found with the Pentacam HR, and poorest with the Orbscan IIz (Table 1). Inter-observer agreement was significantly better in both groups and for all parameters and devices, when the mean of three repeated measurements instead of a single measurement was used (Table 1*, one and mean of three measurements*). This led to a mean reduction of the standard deviations of differences (SD_diff_) of 41% (median, 42.5%, range, 20.8 to 59.1%) across all three parameters and devices. The upper 99.73% LOA with the Pentacam HR with three repeat measurements in keratoconic eyes improved from 1.13 D to 0.57 D for K1 (−49.6%), from 1.93 D to 0.61 D for K2 (−68.4%), from 2.21 D to 1.25 D for Kmax (−43.4%), and from 20.97 μm to 9.93 μm for TCT (−52.6%). As demonstrated for Kmax in the Bland-Altman plots (Fig. 1b), for normal controls and for keratoconus eyes, the variability of differences did not show any dependence on the respective mean values when using either a single (Fig. 1b) or the mean of three repeated measurements (Fig. 1c). Significant measurement bias was found only for TCT readings in healthy eyes with the Casia SS-1000 (SD −0.70 for one and −0.46 for the mean of 3 repeat measurements, respectively, p = 0.04, Supplementary Table S1). Measurement errors appeared not dependent on mean values of K1, K2 and TCT using one measurement and using the mean of three measurements in all devices, for normal controls and for keratoconic eyes (Supplementary Table S1 and Supplementary Figures S4–6).

#### Cut-off values for corneal shape change

The correlation between measurements on the same cornea was found to be small (R = 0.13, Kmax). Estimates for corneal shape change based on 99.73% LOA in keratoconic eyes are outlined in Table 2. For the Pentacam HR, the cut-offs decreased from 2.73 D to 1.55 D for Kmax (−43.2%), from 1.63 D to 0.70 D for K2 (−57.1%), from 1.68 D to 0.75 D for K2 (−55.4%), and from 21.79 μm to 12.12 μm for TCT (−44.8%), when using the mean of three instead of one measurement. For Kmax, a cut-off of 1.55 D implies with 95% confidence, that an increase in Kmax of more than 1.55 D in an eye with KC, taken by two different observers at two different time points, using the mean of three measurements at each time point, theoretically occurs due to measurement error and or inter-observer variability in only 0.135% of imaging sessions.

**Table 2:** Summary of cut-off values for corneal shape change in keratoconic eyes based on one and of mean three repeated measurements, expressed as upper 99.73% LOAs with upper 95% CI.

Device	Parameter	Cut-off for corneal shape change	
		*1 Measurement*	*Mean of 3 measurements*
Pentacam HR	Kmax	2.73	1.55
	K1 (D)	1.63	0.70
	K2 (D)	1.68	0.75
	TCT (μm)	21.97	12.12
Orbscan IIz	K1 (D)	6.60	4.72
	K2 (D)	9.46	5.79
	TCT (μm)	99.30	62.80
Casia SS-1000	K1 (D)	3.77	2.04
	K2 (D)	7.18	2.68
	TCT (μm)	38.39	26.64

#### Interdevice agreement

Agreement between devices was significantly worse than within and between observers using the same device. In keratoconic eyes, agreement was better between the Pentacam and the Casia SS-1000 than between the Pentacam HR and the Orbscan IIz or the Orbscan IIz and the Casia SS-1000 (Supplementary Table S2). There was only slight improvement in agreement between devices when three repeated measurements instead of on measurement were used (Supplementary Table S2), with an overall mean improvement in the of SD_diff_ values of 10.6% (median, 11.2%, range, −7.18 to 28.9%). Cut-off values for corneal change based on three repeat measurements increased drastically between devices compared to the use of Pentacam alone: from 0.70 D to 3.94 (+3.24) D for K1, from 0.75 to 3.79 (+3.04) D, and from 12.12 μm to 51.86 (+39.74) μm, when switching between the Pentacam HR and the Casia SS-1000, and to 4.53 (+3.83) D for K1, to 4.15 (+3.40) D for K2, and to 233.29 (+221.17) μm, when switching between the Pentacam HR and the Orbscan IIz (Supplementary Table S3). Significant relationship between the difference in measurements (bias) and magnitude of measurements were found between the Pentacam HR and the Casia SS-1000 and also between the Orbscan IIz and the Casia SS-1000, evident in the Bland-Altman plots (Supplementary Figures S7–9).

## Discussion

Given the developement and wide clinical use of CXL for KC, corneal topography and tomography is more important than ever in the clinical management of patients with KC. But as with many devices, corneal topographers and tomographers are prone to measurement error: within and between observers, and also between devices. Distinguishing change from measurement error is crucial for the monitoring of KC and planning of treatment. In this study we investigated both theoretically and empirically a method to discriminate change from measurement error in patients with KC, that was also found to be independent on the stage of the disease. Under the assumpion of the independency of three repeated measurements, the mean of three measurements reduces the repeatability coefficient (SD_diff_) by a factor of $\sqrt{3}$ (42.3% reduction), thus narrowing the limits of agreement and consequently improving precision between measurements (*see Supplementary material, Reproducibility analysis*). In our cohort, the repeatability coefficients decreased by a mean of 41.2% (range, 20.8 to 59.1%) compared to coefficients derived from two single measurements; with a corresponding improvement in the LOA. This enabled us to demonstrate that the 99.73% inter-observer LOA based on mean of three repeated measurements were better than the corresponding 95% limits based on one measurement. While such improvement in precision is expected mathematically, to our knowledge no previous studies have demonstrated this reduction in keratoconus or a comparable consistency in improvement of precision.

These findings have important clinical implications for monitoring patients with KC. For example, using the Pentacam HR to measure Kmax, the difference in the mean of three repeated measurements on two different occasions by two different observers will lie outside the 99.73% LOA as a result of measurement variability in only 0.27% of occasions. Hence only 27 out of 20,000 eyes (0.135%) will be mistakenly diagnosed as having progressive disease, if the difference between the means of three repeated measurements for Kmax over two visits differs by more then +1.25 D (95%CI: 0.96D to 1.55 D) D compared to 2.21 D (95%CI: 1.69 D to 2.73 D) when using two single measurements. The combined use of three repeated measurements and 99.73% LOA (upper limits) therefore provides cut-off values to better distinguish between measurement imprecision and corneal change as a marker of progressive disease. Flynn *et al*.^15^ reported upper 95% inter-observer LOA for Kmax of 1.01 D for Pentacam-derived TKC stage 1 or 2, but 3.86 D for stages higher then 2, the latter being greater than the cut-off we propose of 1.51 D using the mean of three measurements and 99.7% LOA.

The use of repeated measurements to improve precision is not new. Epstein *et al*.^23^ compared the variability of the Pentacam using one or five repeated corneal curvature measurements in keratoconic eyes between two visits. In line with our findings, they found the variability to decrease markedly using repeated measurements. Increasing the number of measurements from one to three reduces the LOA by 41%, whereas increasing to five measurements only further reduces the LOA by 14%. Similarly, Hashemi *et al*.^22^ reported a significant improvement in inter-observer agreement for Kmax using the mean of three repeated measurements in patients with KC. We have shown a consistent improvement of LOA across all parameters and devices by a factor of approximately $\sqrt{3}$, as expected. In patients with KC, the 95% limits of inter-observer agreement for Kmax improved from 1.37 D for one measurement to 0.78 D using the mean of three repeated measurements. We validated our results by using a linear mixed model, which showed a consistent reduction of repeatability coefficients for the mean of three measurements in all analyzed parameters (Supplementary Table S4).

The precision of measurements in our study was found to be best for the Pentacam HR, and better in healthy than in keratoconic eyes, which is in line with previous studies^24,25^. Intra-observer agreement was generally better than inter-observer agreement in both groups, which is also consistent with previous studies^15,25^. Although this suggests that measurement precision may be better if patients are scanned by the same observer at each visit, in clinical practice this is not always possible, and hence inter-observer agreement is of more practical importance. We would suggest therefore that in clinical practice with different observers, the mean of three repeated measurements and three, rather than two SD are used to estimate the limits of reproducibility. This provides cut-off values for evidence of change of 0.57 (95%CI: 0.43–0.70) D for K1, 0.61 (95%CI: 0.47–0.75) D for K2, 1.25 (95%CI: 0.96–1.55) D Kmax and 9.93 (95%CI: 7.73–12.12) μm for TCT for patients with KC, irrespective of the severity of disease (within the range tested). The use of three standard deviations increases the specificity of identifying progression by reducing the false-positive rate from 5% down to 0.3%.

We do acknowledge that the practicability of multiple measurements in a clinical setting may not always be accomplished. Precision improves with multiple uncorrelated measurements. The improvement, however, is not linear. There is a large improvement in precision with three measurements and although this improves with further measurements, the gain is modest for the number of additional measurements required. Undertaking three measurements, therefore, significantly improves precision with minimal increase in examination time and inconvenience. Hopefully this inconvenience will be addressed by the instruments’ manufacturers for future device versions. The current Pentacam HR device uses a high speed Scheimpflug camera which is capable of capturing up to 100 scans within a few seconds, and we believe that integrating a serial repeat scan function and adjusting the software for mean calculations of measurements is possible and should be considered in future software updates.

Levels of agreement between devices were relatively poor for all investigated parameters in both keratoconic and healthy eyes. Even using of the mean of three repeated measurements did not improve inter-device precision as much as that seen for the inter-observer precision (mean reduction of repeatability coefficients 13.9% versus 41.2%) (Supplementary Tables S2–3 and Figures S7–9). Switching from the Pentacam HR to either the Casia SS-1000 or the Orbscan IIz is therefore not recommended for monitoring of KC, as this leads to a dramatic decrease in precision. We were not able to compare the same type of device, and further work to determine the precision between two or more of the same type of devices may facilitate monitoring of patients attending different clinical institutions.

There are some limitations to this study. Intra-observer agreement (repeatability) was calculated from two single measurements only, and comparison with the mean of three measurements was therefore not possible. Our study provides no information on the accuracy of the investigated devices due to the absence of a ‘gold standard’. Although the Pentacam HR showed best reproducibility, we do not know if it also provides the most accurate measurements. The image quality limits were established using the Pentacam HR, which may have favoured the performance of the Pentacam compared to the other devices. Not knowing the ‘ground truth’ makes objective quantification of accuracy difficult.

Although the combined use of multiple rather than single tomographic parameters has been put forward to increase sensitivity and specificity to help differentiate between normal and ectatic corneas^17,26,27^, it should be noted that conversely, if the same source meaurements are used to generate multiple indices, these indices will not be independent and the inherent variability in the source measurements will still be reflected and potentially propagated into multiple indices.

Precision is often expressed as 95% limits of agreement (LOA) using 95% confidence intervals. Using three standard deviations (99.7%) together with the mean of three measurements, however, provides an opportunity to improve the specificity of detecting change in patients with KC without reducing the sensitivity. This improves inter-observer precision by 41.2% and provides an acceptable and clinically useful cut-off for evidence of change in patients with KC with only 13.5 false positive out of 10,000 ratings.

## Electronic supplementary material

Supplementary Dataset 1
