# Supplementary material for: Improving precision for detecting change in the shape of the cornea in patients with keratoconus
Source: Sci Rep. 2018 Aug 17;8:12345. doi: 10.1038/s41598-018-30173-7 (PMC6097997; doi:10.1038/s41598-018-30173-7)
Supplement: Supplementary file 1 — Supplementary Dataset 1 [file 41598_2018_30173_MOESM1_ESM.docx]

**Improving precision for detecting change in the shape of the cornea in patients with keratoconus**

**Authors**: Matthias Brunner^1^; Gabriela Czanner^2,4^; Riccardo Vinciguerra^1,3^; Vito Romano^3^; Sajjad Ahmad^1,2^; Mark Batterbury^1^; Claire Britten^1^, Colin E. Willoughby^1,2^; Stephen B. Kaye^1,2^

**Affiliation**:

1. Department of Corneal and External Eye Diseases, St. Paul’s Eye Unit, Royal Liverpool University Hospital, Liverpool, United Kingdom
2. Department of Eye and Vision Science, University of Liverpool, Liverpool, United Kingdom
3. Department of Surgical Sciences, Division of Ophthalmology, University of Insubria, Varese, Italy
4. Department of Biostatistics, University of Liverpool, Liverpool, United Kingdom

**Supplementary Material**

**1. Reproducibility analysis in devices using first measurement and the mean of** $\boldsymbol{m}$ **repeat measurements from two observers**

Obtaining three measurements rather than just one is a feasible protocol. Intuitively, the mean of three measurements should have a smaller variance. Mathematically, the variance of the mean of 3 independent measurements is lower by factor of three and hence the variance of difference of two independent means is also lower by factor of 3.^1,2^ If the 3 measurements are not independent, the variance does not decrase as much.

The precision of the mean of $m$ measurements (of e.g. Kmax) is derived in two steps as follows.^2^ First, we assume we have $m$ measurements done by 2 observers. The variability of measurements is then made up of three components: a) the variability across individual corneas of the true quantity being measured, b) the variability of each individual’s mean values about overall mean for that observer (also called heterogeneity), and c) the variability of repeated measurements about the mean for an individual cornea (also called measurement error). We assume that the component c) is constant across all corneas and both observers $SD_{w}$. The values of all components can be obtained by performing 2-way ANOVA with two fixed factors: one factor for eye (to account for the component a) and one factor for the grader (to account for component b). Then the residual standard error from the ANOVA is the estimate of $SD_{w}$i.e. of the component c). Bland-Altman^3^ proposed to run separate 1-way ANOVAs for each of graders and then to combine the standard errors, which is also a suitable method.

Secondly, under the assumption of ***independent*** repeated measurements from a patient obtained by one observer, the SD of the within-eye average of $m$ measurements can be obtained as the standard error of the mean, which is

$1/\sqrt{m}\times SD_{w}$;

where $SD_{w}$ is standard deviation of single measurement from the patient. Then the SD of the difference of two sample means from same eye is (Bland and Altman)

$$1/\sqrt{m}\times SD_{diff}=\sqrt{2}/\sqrt{m}\times SD_{w}.$$

This has an important implication for monitoring of disease because the coefficient of repeatability for a mean of $m$ repeated measurements is also $\sqrt{m}$ times smaller and can be calculated as follows

$CR=u_{1-\alpha/2}\times\sqrt{2/m}\times SD_{w}$,

where $m$ is the number of repeated measurements and $u_{1-\alpha/2}$ is the $\left( 1-\alpha/2 \right)\%$ quantile of the standard Normal distribution. Hence a coefficient of repeatability of a mean of $m$ repeated measurements is $\sqrt{m}$ times smaller than the coefficient of repeatability of single measurements.

For example, in our study for Kmax of the Pentacam, the standard deviations of differences between observers using a single measurement were 0.21 D and 0.81 D for healthy and keratoconic eyes, respectively, i.e. $\sqrt{2}\times SD_{w}$= 0.21 D and 0.81 D. Using the difference of the mean of 3 measurements, the standard deviation reduced to 0.11 D and 0.46 D for normal and keratoconic eyes, respectively. This is consistent with the formulae above, that is,

0.21/$\sqrt{3}$=0.12≈0.11 D

0.81/$\sqrt{3}$=0.47≈0.46 D.

This suggests that the measurements done by an observer are independent i.e. there is no interaction between measurement error and observer, which is what is expected if measurements are independent. The independency of measurements can be tested via a correlation coefficient (e.g. in linear mixed effect model). If the correlation coefficient is zero and if data are normally distributed, then this means that the measurements errors are independent.

In our study we calculated the LOA for mean of repeated measurements in two ways and systematically compared them. We used a traditional approach, where we calculated the mean (of the three measurements) for each eye and then evaluated the agreement in the means via Bland and Altman's method.^4^ In our second approach we used a 2-way ANOVA with two fixed factors (as described above), where one factor is for the eye and one factor for the observer. We obtained same standard deviations (hence same LOA) results in both methods in four corneal parameters (K1, K2, Kmax and TCT) derived from two single as well as the mean of three repeat measurements from three different corneal topo- and tomography devices (Pentacam HR, Casia SS-100, and Orbscan II) *(Supplementary material, Table 3 and 4)*. Another approach is the linear mixed effect model used by Epstein et al. 2012^5^, however, this does not take into account multiple observers which is important in clinical practice.

Various factors, including the use of different statistical methods and clinical charactersitics of study subjects, may lead to discrepancies of repeatability and reproducibility and render it difficult to compare data between studies. Selection of appropriate statistical methods is critical for precision analysis. The quantification of agreement, as proposed by Altman and Bland^4^, is a well established and widely used method for the estimation of measurement precision and comparison of measurement methods. It relates the magnitude of the measurement errors to the inherent variability of the quantity of interest (reliability).^6^

Precision analyses of a single measurement are typically based on one measurement taken by each of two observers and they provide a useful repeatability coefficient defined as two times the standard deviation of the difference (SD_diff_) between the two measurements (i.e. 95% LOA=2x SD_diff_).^6^ Until now, no clear framework has been agreed on how to quantify agreement when 2 or more repeated measurements are obtained for each eye by each of two observers. The method used in this study provides a solution to evaluation of precision in repeated measurements studies. First, we demonstrate (propose) that a two-way ANOVA must be used for the calculation of the within-eye measurement variability (SD_W_) and of the bias between observers. Second, we propose that a Bland-Altman plot is created for pairs of measurements to evaluate any deviations from Normal distribution (as LOA value assumes Normal distribution of the differences, and hence it should not be trusted without judging the assumptions via the Bland-Altman plot). Third, correlations between measurements need to be tested and confirmed to be zero *(Supplementary material, Table 6)*.

References

1. Bland JM, Altman DG. Measurement error proportional to the mean. BMJ 1996;313:106.
2. Bland JM, Altman DG. Agreement between methods of measurement with multiple observations per individual. J Biopharm Stat 2007;17:571–582.
3. Bland JM, Altman DG. Statistics notes: Measurement error. BMJ 1996;312:1654–1654.
4. Bland JM, Altman DG. Statistical methods for assessing agreement between two methods of clinical measurement. Lancet 1986;1:307–310.
5. Epstein RL, Chiu Y-L, Epstein GL. Pentacam HR Criteria for Curvature Change in Keratoconus and Postoperative LASIK Ectasia. J Refract Surg 2012;28:890–894.
6. Bartlett JW, Frost C. Reliability, repeatability and reproducibility: analysis of measurement errors in continuous variables. Ultrasound Obstet Gynecol 2008;31:466–475.

**2. Supplementary Figures**

**
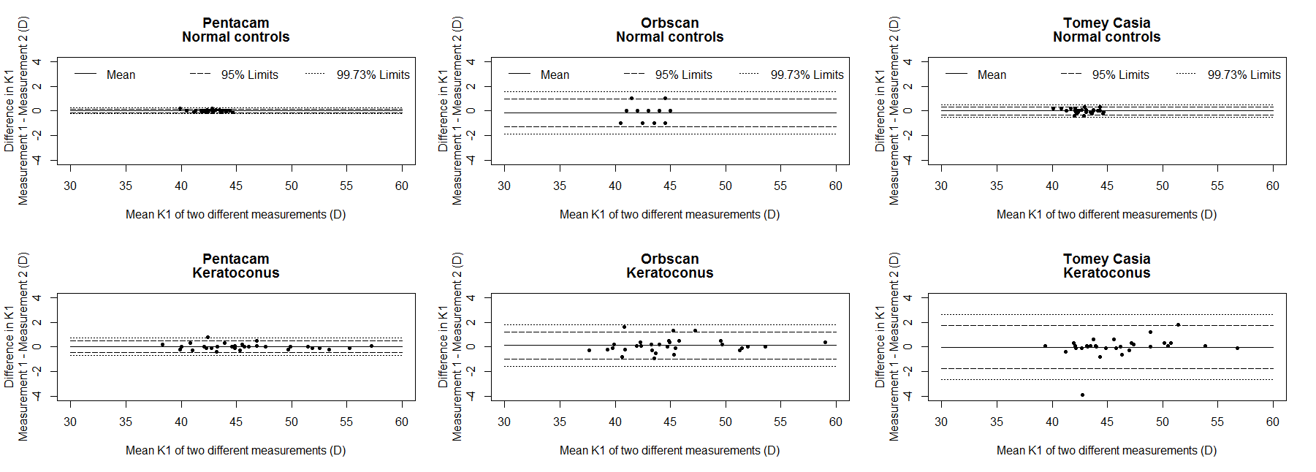
**

**Figure S1.** Bland-Altman plots for intra-observer 95% and 99.73% limits of agreement of K1 in healthy and keratoconic eyes (n=30).


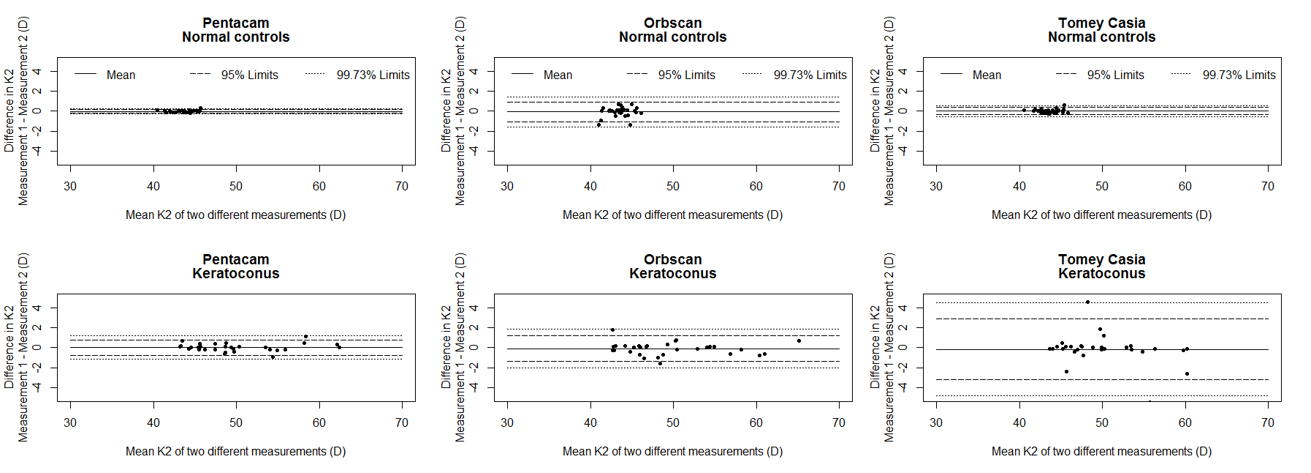


**Figure S2.** Bland-Altman plots for intra-observer 95% and 99.73% limits of agreement of K2 in healthy and keratoconic eyes (n=30).

**
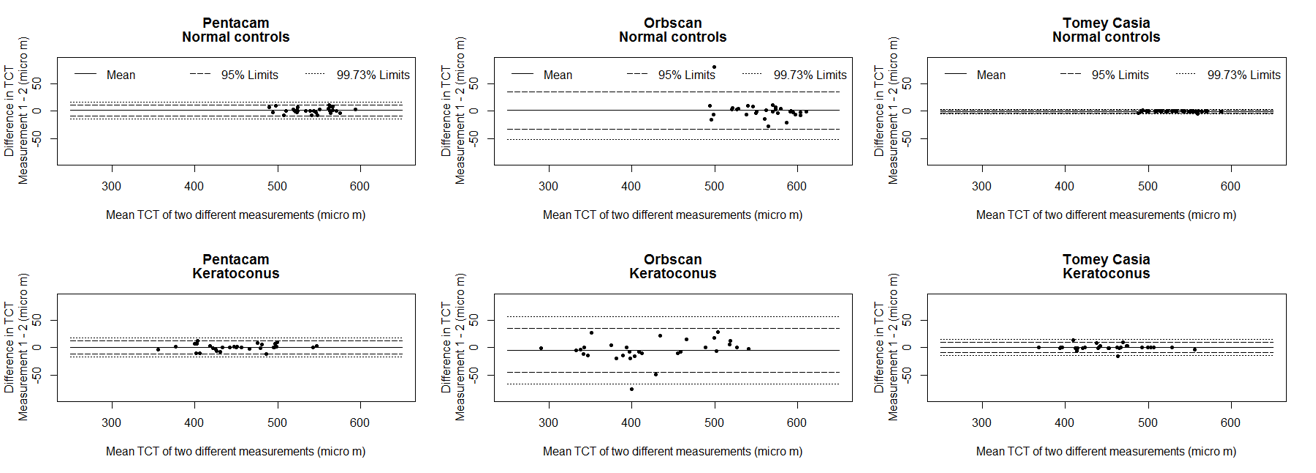
**

**Figure S3.** Bland-Altman plots for intra-observer 95% and 99.73% limits of agreement of TCT in healthy and keratoconic eyes (n=30).


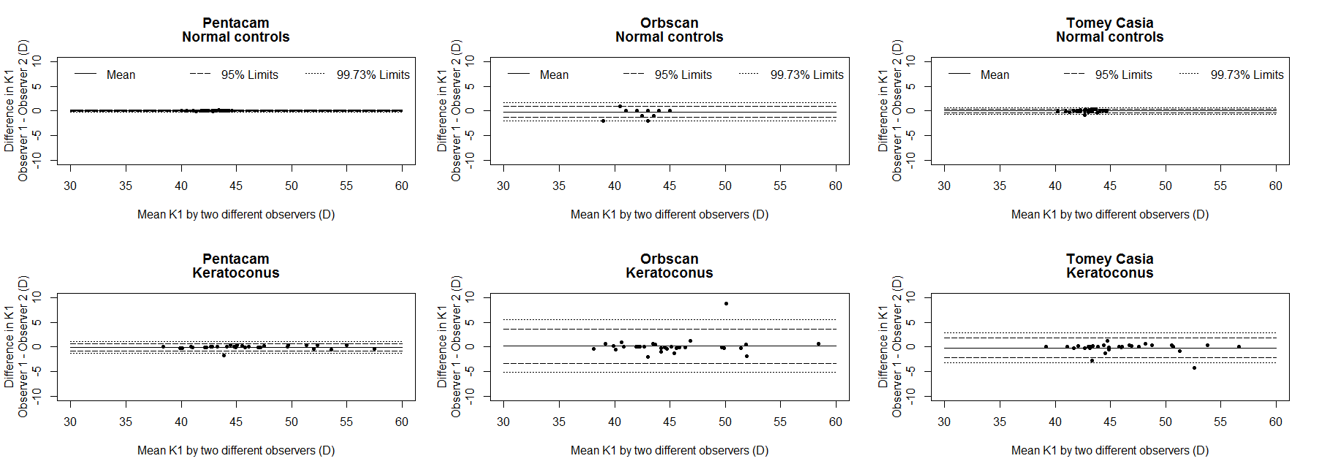


**Figure S4.** Bland-Altman plots for inter-observer 95% and 99.73% limits of agreement of K1 by two different observers in healthy and keratoconic eyes (n=30).

**
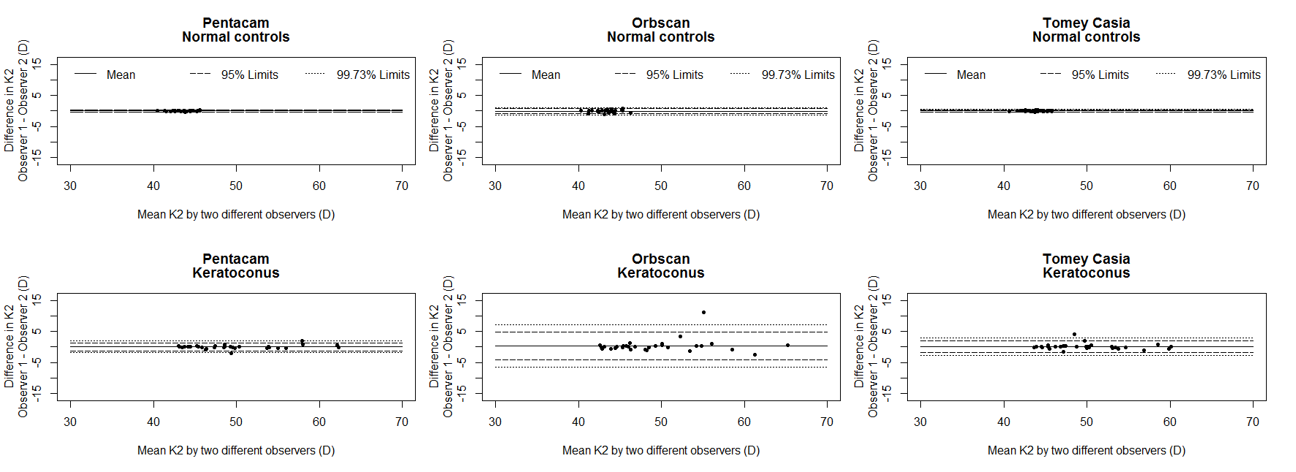
**

**Figure S5.** Bland-Altman plots for inter-observer 95% and 99.73% limits of agreement of K2 by two different observers in healthy and keratoconic eyes (n=30).

**
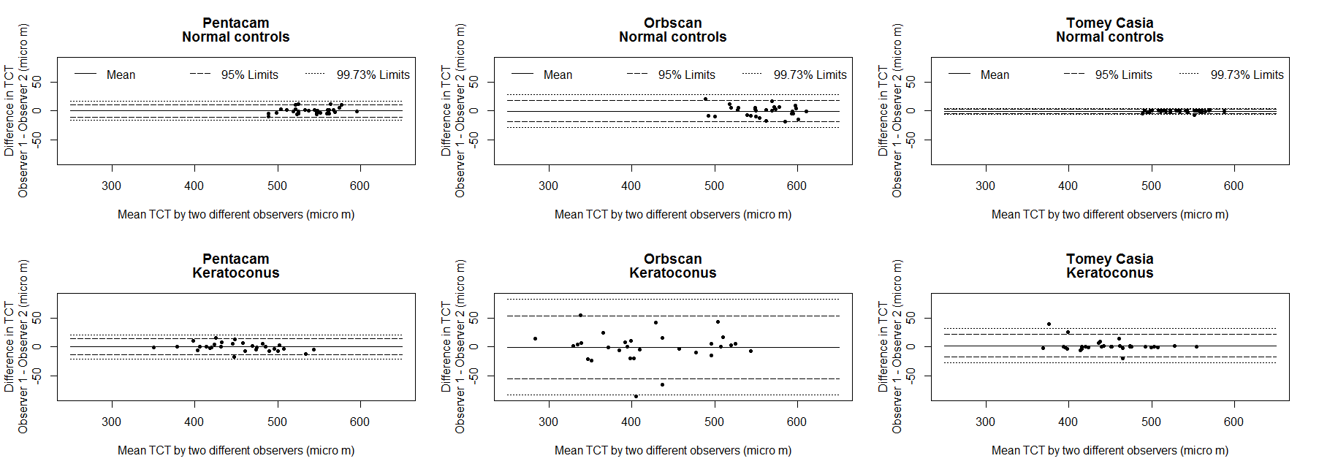
**

**Figure S6.** Bland-Altman plots for inter-observer 95% and 99.73% limits of agreement of TCT by two different observers in healthy and keratoconic eyes (n=30).


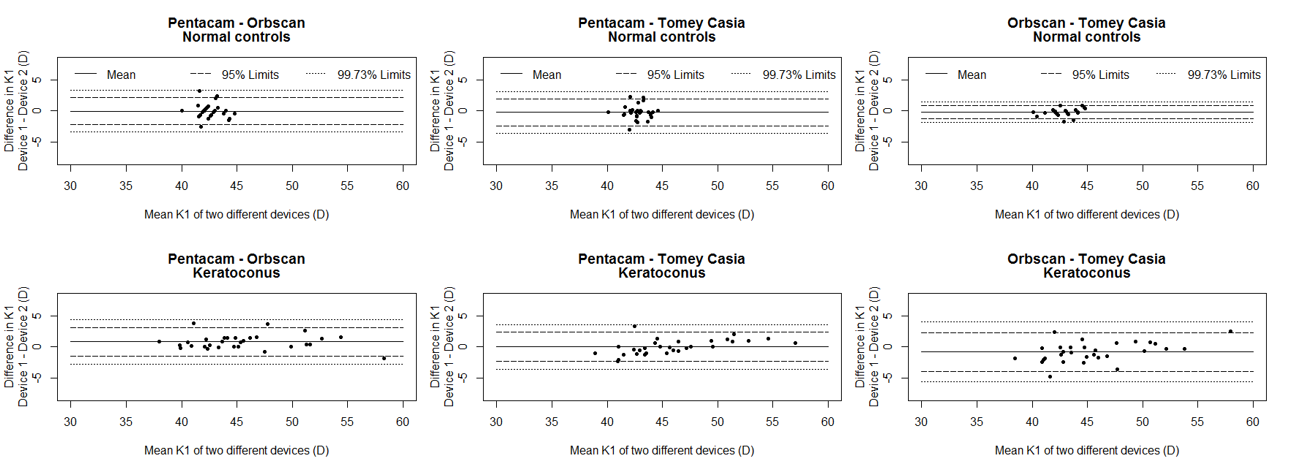


**Figure S7.** Bland-Altman plots for inter-device 95% and 99.73% limits of agreement of K1 by two different devices in healthy and keratoconic eyes (n=30).

**
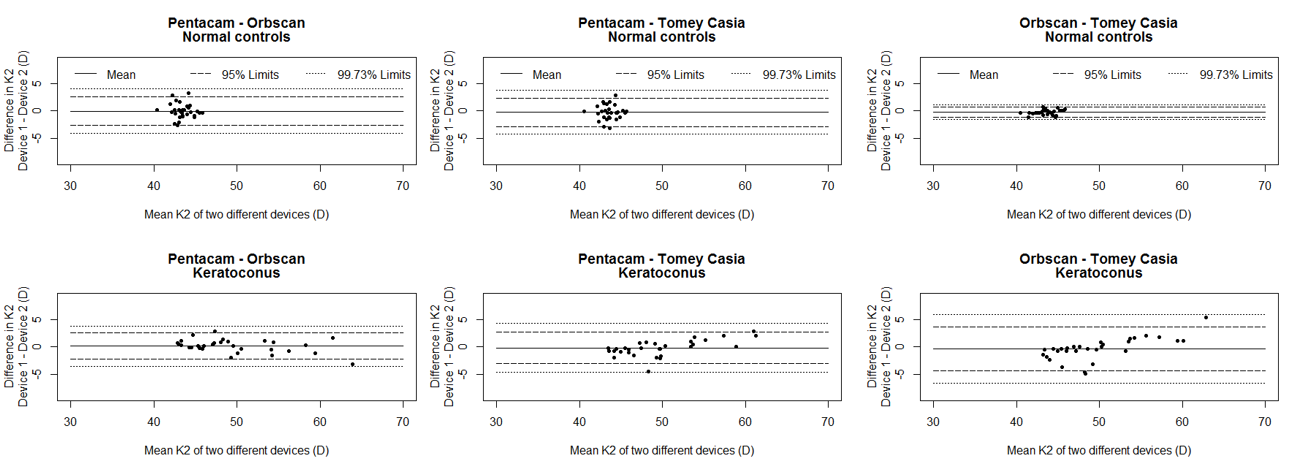
**

**Figure S8.** Bland-Altman plots for inter-device 95% and 99.73% limits of agreement of K2 by two different devices in healthy and keratoconic eyes (n=30).

**
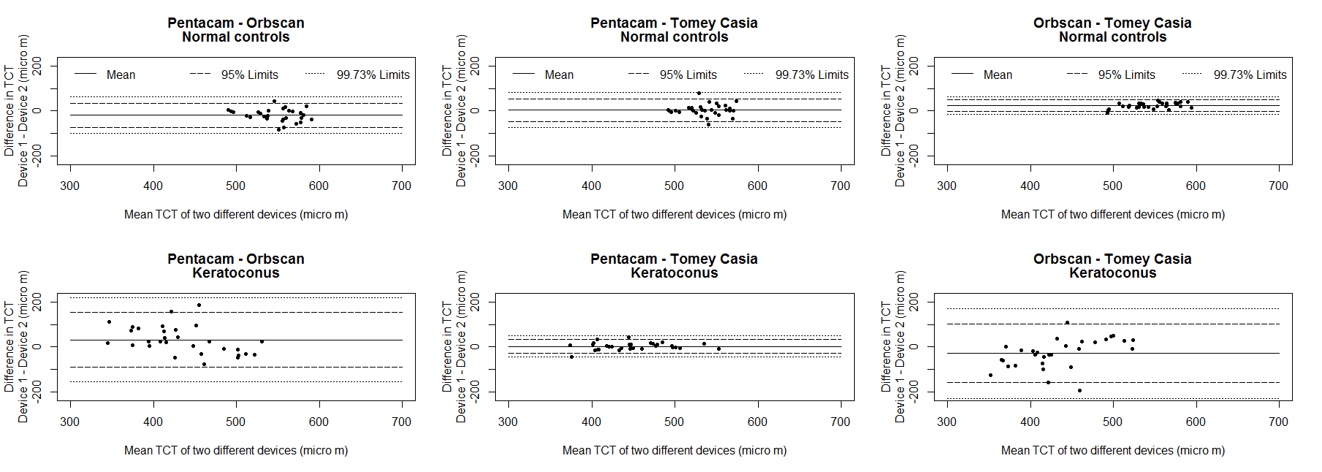
**

**Figure S9.** Bland-Altman plots for inter-device 95% and 99.73% limits of agreement of TCT by two different devices in healthy and keratoconic eyes (n=30).

**3. Supplementary Tables**

**Table S1.** Inter-observer 95% and 99.73% limits agreement (first-second observer), standard deviations of the difference, and measurement bias for K1, K2, and TCT in three devices for first measurement and mean of three measurements from each observer in healthy and keratoconic eyes.

| **Parameter** | **No. of Measurements** | **Agreement** | **Pentacam HR** | | **Orbscan IIz** | | **Casia SS-1000** | |
| --- | --- | --- | --- | --- | --- | --- | --- | --- |
|  |  |  | *Controls* | *Keratoconus* | *Controls* | *Keratoconus* | *Controls* | *Keratoconus* |
| K1 (D) | 1* | Bias (p-value)* | 0.02 (p=0.17) | -0.05 (p=0.46) | -0.17 (p=0.59) | 0.18 (p=0.57) | -0.01 (p=0.81) | -0.18 (p=0.34) |
|  |  | SD | 0.06 | 0.39 | 0.59 | 1.76 | 0.22 | 1.01 |
|  |  | 95% LOA | -0.11 to 0.12 | -0.82 to 0.72 | -1.33 to 0.99 | -3.26 to 3.63 | -0.44 to 0.43 | -2.16 to 1.81 |
|  |  | 99.73% LOA | -0.18 to 0.21 | -1.23 to 1.13 | -1.94 to 1.61 | -5.09 to 5.46 | -0.68 to 0.66 | -3.22 to 2.86 |
|  |  |  |  |  |  |  |  |  |
|  | Mean of 3** | Bias (p-value)* | 0.01 (p=0.24) | -0.06 (p=0.10) | -0.07 (p=0.23) | 0.27 (p=0.24) | 0.01 (p=0.70) | -0.07 (p=0.50) |
|  |  | SD | 0.04 | 0.21 | 0.30 | 1.22 | 0.09 | 0.58 |
|  |  | 95% LOA | -0.06 to 0.08 | -0.48 to 0.35 | -0.65 to 0.51 | -2.13 to 2.66 | -0.17 to 0.19 | -1.21 to 1.06 |
|  |  | 99.73% LOA | -0.10 to 0.11 | -0.70 to 0.57 | -0.95 to 0.82 | -3.40 to 3.93 | -0.27 to 0.28 | -1.81 to 1.67 |
|  |  |  |  |  |  |  |  |  |
| K2 (D) | 1 | Bias (p-value)* | -0.02 (p=0.34) | 0.01 (p=0.93) | -0.08 (p=0.30) | *0.38 (p=0.36)* | 0.01 (p=0.66) | 0.13 (p=0.46) |
|  |  | SD | 0.13 | 0.64 | 0.4 | 2.28 | 0.17 | 0.96 |
|  |  | 95% LOA | -0.28 to 0.23 | -1.25 to 1.27 | -0.85 to 0.70 | -4.08 to 4.84 | -0.31 to 0.34 | -1.75 to 2.01 |
|  |  | 99.73% LOA | -0.41 to 0.37 | -1.91 to 1.93 | -1.27 to 1.11 | -6.45 to 7.22 | -0.48 to 0.51 | -2.75 to 3.01 |
|  |  |  |  |  |  |  |  |  |
|  | Mean of 3 | Bias (p-value)* | -0.02 (p=0.18) | -0.05 (p=0.26) | -0.09 (p=0.04) | 0.19 (p=0.49) | <0.01 (p=0.99) | -0.10 (p=0.46) |
|  |  | SD | 0.07 | 0.32 | 0.23 | 1.54 | 0.10 | 0.76 |
|  |  | 95% LOA | -0.15 to 0.11 | -0.67 to 0.58 | -0.53 to 0.36 | -2.82 to 3.20 | -0.19 to 0.19 | -1.60 to 1.39 |
|  |  | 99.73% LOA | -0.22 to 0.18 | -1.01 to 0.91 | -0.77 to 0.59 | -4.41 to 4.80 | -0.29 to 0.29 | -2.39 to 2.19 |
|  |  |  |  |  |  |  |  |  |
| TCT (μm) | 1 | Bias (p-value)* | 0.33 (p=0.74) | 0.03 (p=0.98) | -0.27 (p=0.88) | -0.63 (p=0.90) | -0.70 (p=0.04) | 2.30 (p=0.21) |
|  |  | SD | 5.24 | 6.98 | 9.37 | 27.40 | 1.78 | 9.90 |
|  |  | 95% LOA | -10.29 to 10.96 | -13.65 to 13.71 | -18.64 to 18.10 | -54.34 to 53.08 | -4.20 to 2.80 | -17.10 to 21.70 |
|  |  | 99.73% LOA | -15.93 to 16.61 | -20.91 to 20.97 | -28.39 to 27.85 | -82.84 to 81.58 | -6.05 to 4.65 | -27.39 to 31.99 |
|  |  |  |  |  |  |  |  |  |
|  | Mean of 3 | Bias (p-value)* | -0.12 (p=0.81) | -0.24 (p=0.70) | 0.60 (p=0.51) | -0.06 (p=0.99) | -0.46 (p=0.04) | 0.81 (p=0.54) |
|  |  | SD | 2.70 | 3.39 | 4.91 | 17.24 | 1.18 | 7.08 |
|  |  | 95% LOA | -5.42 to 5.18 | -6.88 to 6.40 | -9.01 to 10.21 | -33.84 to 33.73 | -2.76 to 1.85 | -13.08 to 14.69 |
|  |  | 99.73% LOA | -8.24 to 7.99 | -10.42 to 9.93 | -14.12 to 15.32 | -51.76 to 51.65 | -3.98 to 3.07) | -20.44 to 22.06 |
| Bias=Mean difference, SD=Standard deviation of difference, LOA=Limits of agreement; | | | | | |  |  |  |
| * Paired t-test for statistical significance of the bias (i.e. H0: mean difference=0) | | | | |  |  |  |  |

**Table S2.**  Inter-device 95% and 99.73% limits of agreement, standard deviations of the difference, and measurement bias for K1, K2, and TCT for first measurement and mean of three measurements from each observer in healthy and keratoconic eyes.

| **Parameter** | **No. of Measurements** | **Agreement** | **Pentacam - Orbscan** | | **Pentacam - Casia** | | **Orbscan - Casia** | |
| --- | --- | --- | --- | --- | --- | --- | --- | --- |
|  |  |  | *Controls* | *Keratoconus* | *Controls* | *Keratoconus* | *Controls* | *Keratoconus* |
| K1 (D) | 1** | Bias (p-value)* | -0.01 (p=0.95) | 0.83 (p<0.01) | -0.21 (p=0.31) | 0.03 (p=0.91) | -0.20 (p=0.06) | -0.80 (p=0.01) |
|  |  | SD | 1.13 | 1.19 | 1.12 | 1.20 | 0.56 | 1.61 |
|  |  | 95% LOA | -2.23 to 2.21 | -2.23 to 2.21 | -2.40 to 1.98 | -2.33 to 2.39 | -1.29 to 0.89 | -3.95 to 2.35 |
|  |  | 99.73% LOA | -3.41 to 3.39 | -2.73 to 4.38 | -3.57 to 3.15 | -3.58 to 3.64 | -1.87 to 1.47 | -5.62 to 4.01 |
|  |  |  |  |  |  |  |  |  |
|  | Mean of 3*** | Bias (p-value)* | -0.04 (p=0.82) | 0.75 (p<0.01) | -0.02 (p=0.34) | -0.08 (p=0.69) | -0.16 (p=0.04) | -0.82 (p<0.01) |
|  |  | SD | 1.08 | 1.04 | 0.34 | 1.02 | 0.4 | 1.37 |
|  |  | 95% LOA | -2.17 to 2.08 | -1.29 to 2.78 | -2.43 to 2.02 | -2.07 to 1.92 | -0.93 to 0.62 | -3.52 to 1.87 |
|  |  | 99.73% LOA | -3.29 to 3.20 | -2.37 to 3.86 | -3.61 to 3.20 | -3.13 to 2.98 | -1.35 to 1.03 | -4.95 to 3.31 |
|  |  |  |  |  |  |  |  |  |
| K2 (D) | 1** | Bias (p-value)* | -0.02 (p=0.94) | 0.17 (p=0.44) | -0.25 (p=0.32) | *-0.17 (p=0.53)* | -0.23 (p=0.01) | -0.35 (p=0.37) |
|  |  | SD | 1.34 | 1.22 | 1.33 | 1.48 | 0.45 | 2.06 |
|  |  | 95% LOA | -2.65 to 2.61 | -2.21 to 2.56 | -2.85 to 2.36 | -3.07 to 2.72 | -1.11 to 0.65 | -4.39 to 3.70 |
|  |  | 99.73% LOA | -4.04 to 4.00 | -3.48 to 3.83 | -4.23 to 3.74 | -4.60 to 4.25 | -1.57 to 1.12 | -6.54 to 5.84 |
|  |  |  |  |  |  |  |  |  |
|  | Mean of 3*** | Bias (p-value)* | -0.38 (p=0.87) | -0.02 (p=0.92) | -0.23 (p=0.33) | -0.29 (p=0.17) | -0.20 (p<0.01) | -0.27 (p=0.41) |
|  |  | SD | 1.30 | 1.14 | 1.30 | 1.12 | 0.32 | 1.74 |
|  |  | 95% LOA | -2.59 to 2.51 | -2.26 to 2.22 | -2.79 to 2.32 | -2.48 to 1.91 | -0.82 to 0.42 | -3.67 to 3.14 |
|  |  | 99.73% LOA | -3.95 to 3.87 | -3.45 to 3.41 | -4.14 to 3.67 | -3.64 to 3.07 | -1.15 to 0.75 | -5.48 to 4.95 |
|  |  |  |  |  |  |  |  |  |
| TCT (μm) | 1** | Bias (p-value)* | -19.37 (p<0.01)* | 31.57 (p<0.01) | 4.30 (p=0.36) | 2.73 (p=0.35) | 23.67 (p<0.01) | -28.83 (p=0.02) |
|  |  | SD | 27.18 | 62.53 | 25.74 | 16.00 | 12.95 | 66.33 |
|  |  | 95% LOA | -72.64 to 33.91 | -91.00 to 154.13 | -46.15 to 54.75 | -28.62 to 34.08 | -1.72 to 49.0 | -158.84 to 101.17 |
|  |  | 99.73% LOA | -100.91 to 62.17 | -156.04 to 219.17 | -72.91 to 81.51 | -45.25 to 50.72 | -15.18 to 62.52 | -227.82 to 170.15 |
|  |  |  |  |  |  |  |  |  |
|  | Mean of 3*** | Bias (p-value)* | -19.63 (p<0.01) | 27.79 (p=0.01) | 3.89 (p=0.41) | 1.96 (p=0.44) | 23.52 (p<0.01) | -25.83 (p=0.02) |
|  |  | SD | 27.57 | 56.35 | 25.4 | 13.68 | 13.88 | 61.08 |
|  |  | 95% LOA | -73.68 to 34.41 | -82.66 to 138.24 | -45.90 to 53.68 | -24.87 to 28.78 | 3.69 to 50.74 | -145.56 to 93.89 |
|  |  | 99.73% LOA | -102.35 to 63.09 | -141.26 to 196.84 | -72.31 to 80.09 | -39.09 to 43.01 | -18.13 to 65.17 | -209.08 to 157.42 |
| Bias=Mean difference, SD=Standard deviation of difference, LOA=Limits of agreement; | | | | | |  |  |  |
| * Paired t-test for statistical significance of the bias (i.e. H0: mean difference=0) | | | | |  |  |  |  |

**Table S3.** Comparison of within- and inter-device cut-off values for corneal shape change

|  | **Cut-offs* when using same device between observers** | | |
| --- | --- | --- | --- |
| Parameter | Pentacam HR | Casia SS-1000 | Orbscan IIz |
| K1 | 0.70 | 2.04 | 4.72 |
| K2 | 0.75 | 2.68 | 5.79 |
| TCT | 12.12 | 26.64 | 62.80 |
|  |  |  |  |
|  | **Increase of cut-offs* when switching between devices** | | |
| Paramter | Pentacam-Casia | Pentacam-Orbscan | Orbscan-Casia |
| K1 (D) | 3.94^†^ (+3.24) | 4.53 (+3.83) | 4.19^†^ (+0.53) |
| K2 (D) | 3.79^†^ (+3.04) | 4.15 (+3.4) | 6.07^†^ (+0.28) |
| TCT (μm) | 51.86 (+39.74) | 233.29 (+221.17) | 196.92 (+134.12) |
| *Derived from 99.97% interobserver LOA and 3 measurements | | |  |
| ^†^The cutoff depends on the value of the measurement | | |  |

**Table S4.** Comparison of inter-observer 95% and 99.73% limits of agreement for Kmax for first measurement and mean of three measurements from each observer in healthy and keratoconic eyes using the standard method and 2-way ANOVA.

| **Parameter** | **No. of Measurements** | **Agreement** | **Pentacam** | |  |
| --- | --- | --- | --- | --- | --- |
|  |  |  | *Controls* | *Keratoconus* | **Method** |
| Kmax | 1 | Diff (p-value)* | -0.01 (p=0.80) | -0.21 (p=0.17) | Standard method, |
|  |  | SD | 0.21 | 0.81 | using first measurements of observers 1 and 2 |
|  |  | 95% LOA (1.96 SD) | -0.43 to 0.41 | -1.56 to 1.30 |  |
|  |  | 99.73% LOA (3 SD) | -0.63 to 0.65 | -2.35 to 2.07 |  |
|  |  |  |  |  |  |
|  |  |  |  |  | 2-way ANOVA with factor eye and observer, |
|  |  | SD | 0.21 | 0.81 | using first measurement of observers 1 and 2 |
|  |  |  |  |  |  |
|  |  |  |  |  |  |
|  |  |  |  |  |  |
|  | Mean of 3 | Diff (p-value)* | -0.02 (p=0.35) | -0.11 (p=0.18) | Standard method, |
|  |  | SD | 0.11 | 0.46 | Using all **three measurements** of observer 1 and 2 |
|  |  | 95% LOA | -0.23 to 0.20 | -1.01 to 0.78 |  |
|  |  | 99.73% LOA | -0.35 to 0.31 | -1.48 to 1.25 |  |
|  |  |  |  |  |  |
|  |  |  |  |  | 2-way ANOVA with factor eye and observer, |
|  |  | SD | 0.11 | 0.43 | using all **three measurements** of observers 1 and 2 |
|  |  |  |  |  |  |
|  |  |  |  |  |  |
| Diff=Mean difference, SD=Standard deviation of difference, LOA=limits of agreement. | | | | | |
| *Paired t-test for statistical significance of the difference (i.e. H0: mean difference=0). | | | | | |
|  | | | | | |
